# KATNAL1 Regulation of Sertoli Cell Microtubule Dynamics Is Essential for Spermiogenesis and Male Fertility

**DOI:** 10.1371/journal.pgen.1002697

**Published:** 2012-05-24

**Authors:** Lee B. Smith, Laura Milne, Nancy Nelson, Sharon Eddie, Pamela Brown, Nina Atanassova, Moira K. O'Bryan, Liza O'Donnell, Danielle Rhodes, Sara Wells, Diane Napper, Patrick Nolan, Zuzanna Lalanne, Michael Cheeseman, Josephine Peters

**Affiliations:** 1 MRC Centre for Reproductive Health, University of Edinburgh, The Queen's Medical Research Institute, Edinburgh, United Kingdom; 2 Institute of Experimental Morphology and Anthropology with Museum, Bulgarian Academy of Sciences, Sofia, Bulgaria; 3 The Department of Anatomy and Developmental Biology, Monash University, Melbourne, Victoria, Australia; 4 Prince Henry's Institute of Medical Research, Clayton, Victoria, Australia; 5 Mary Lyon Centre, Medical Research Council, Harwell Science and Innovation Campus, Harwell, Oxfordshire, United Kingdom; 6 Mammalian Genetics Unit, Medical Research Council, Harwell Science and Innovation Campus, Harwell, Oxfordshire, United Kingdom; The Jackson Laboratory, United States of America

## Abstract

Spermatogenesis is a complex process reliant upon interactions between germ cells (GC) and supporting somatic cells. Testicular Sertoli cells (SC) support GCs during maturation through physical attachment, the provision of nutrients, and protection from immunological attack. This role is facilitated by an active cytoskeleton of parallel microtubule arrays that permit transport of nutrients to GCs, as well as translocation of spermatids through the seminiferous epithelium during maturation. It is well established that chemical perturbation of SC microtubule remodelling leads to premature GC exfoliation demonstrating that microtubule remodelling is an essential component of male fertility, yet the genes responsible for this process remain unknown. Using a random ENU mutagenesis approach, we have identified a novel mouse line displaying male-specific infertility, due to a point mutation in the highly conserved ATPase domain of the novel KATANIN p60-related microtubule severing protein Katanin p60 subunit A-like1 (KATNAL1). We demonstrate that *Katnal1* is expressed in testicular Sertoli cells (SC) from 15.5 days post-coitum (dpc) and that, consistent with chemical disruption models, loss of function of KATNAL1 leads to male-specific infertility through disruption of SC microtubule dynamics and premature exfoliation of spermatids from the seminiferous epithelium. The identification of KATNAL1 as an essential regulator of male fertility provides a significant novel entry point into advancing our understanding of how SC microtubule dynamics promotes male fertility. Such information will have resonance both for future treatment of male fertility and the development of non-hormonal male contraceptives.

## Author Summary

To identify new genes involved in male fertility, we have used a chemical treatment to induce random mutations within the DNA of mice and then screened for mice exhibiting male infertility. We then used genetic mapping techniques to identify which chromosome the responsible gene was situated within and DNA sequencing to identify the mutation, which was found in *Katnal1*. We tested the mutated copy of the gene in cells and determined it to be non-functional. We then used antibody detection to determine that, within the testis, KATNAL1 protein is expressed in the sperm-cell supporting Sertoli cells in both mice and humans. Examination of testicular tissue revealed that KATNAL1 functions to control Sertoli cell microtubule dynamics and retention of sperm during their maturation within the tubules of the testis; absence of KATNAL1 results in premature release of immature sperm and male infertility. The identification and characterisation of this gene, the first microtubule severing protein shown to function in Sertoli cells, will further our understanding of how male fertility is promoted, while also providing important information relevant to the development of male contraceptives.

## Introduction

The testis is a complex multi-cellular organ and it has long been known that cell-cell interactions between somatic and germ cells are essential for normal testis function [1]. During spermatogenesis, the somatic Sertoli cells (SCs), specialised polarised epithelial cells, form junctional complexes with germ cells and act to protect and support germ cells physically, metabolically, and immunologically during their maturation from diploid spermatogonia, through meiosis and during remodelling into mature spermatozoa [2], [3]. The supporting roles undertaken by SCs are facilitated through an active cytoskeleton, with parallel arrays of microtubules arranged radially along the polarised luminal-basement membrane axis [4], with their minus ends directed towards the apical surfaces [5], [6]. These microtubules are involved in maintaining the shape of the highly branched and dynamic Sertoli cells, transporting and positioning organelles in the cytoplasm, and secreting seminiferous tubule fluid. Furthermore, microtubule-based transport machinery is coupled to intercellular junctions to ensure retention of spermatids to SCs, whilst also permitting translocation of spermatids in the seminiferous epithelium during remodelling (for review see [7]).

Systemic treatment with chemical inhibitors of microtubule assembly such as colchicine, [8], [9], or promoters of microtubule assembly such as 2–5-Hexanedione [10] both induce germ cell loss from the seminiferous epithelium. Furthermore, studies specifically targeting microtubules within SCs, such as SC-specific over-expression of the microtubule nucleating protein, gamma-tubulin [11], [12], or SC-specific expression of a dominant-negative form of the microtubule plus end binding protein EB1 [13] induce similar germ cell loss from the seminiferous epithelium. Together these studies demonstrate that SC microtubule-dependent functions are extremely sensitive to aberrant microtubule remodelling. However, despite our detailed understanding of the significant roles microtubules play within the SCs to support male fertility, the molecular mechanisms that facilitate microtubule remodelling within SCs remain largely unknown.

Some inferences can be made through comparison to analogous systems elsewhere in the body. The SC cytoskeleton shares morphological, structural and functional properties with the neuronal cytoskeleton [4]. Within neurones, microtubule remodelling is facilitated *in vivo* by the ATPase microtubule severing proteins, SPASTIN [14], [15] and KATANIN p60 [16]–[20]. These function to permit neuronal plasticity by controlling axonal growth through disruption of the microtubule lattice in an ATP-dependent reaction [16], [17], [20],[21]. Given the similarities in cytoskeleton structure and plasticity between the cell-types it is logical to hypothesise the existence of a similar mechanism controlling microtubule remodelling within SCs.

A novel ATPase protein has been named Katanin p60 subunit A-like1 (KATNAL1) when annotated in Ensembl (www.ensembl.org: ENSMUSG00000041298), based upon its sequence homology to KATANIN p60 (66% identity, 78% conserved). Furthermore, recent *in silico* profile-profile matching and *ab initio* structure modelling using ROSETTA (www.rosettadesigngroup.com) [22], along with recent over-expression studies in cell-lines [23], [24] strongly suggests that KATNAL1 has a similar microtubule-severing role to KATANIN p60. In this paper we describe the identification of a mouse line homozygous for an ENU-induced null allele of *Katnal1*, and detail the first *in vivo* function ascribed to this novel microtubule severing protein.

## Results

### Identification of infertile males

**Figure 1:**
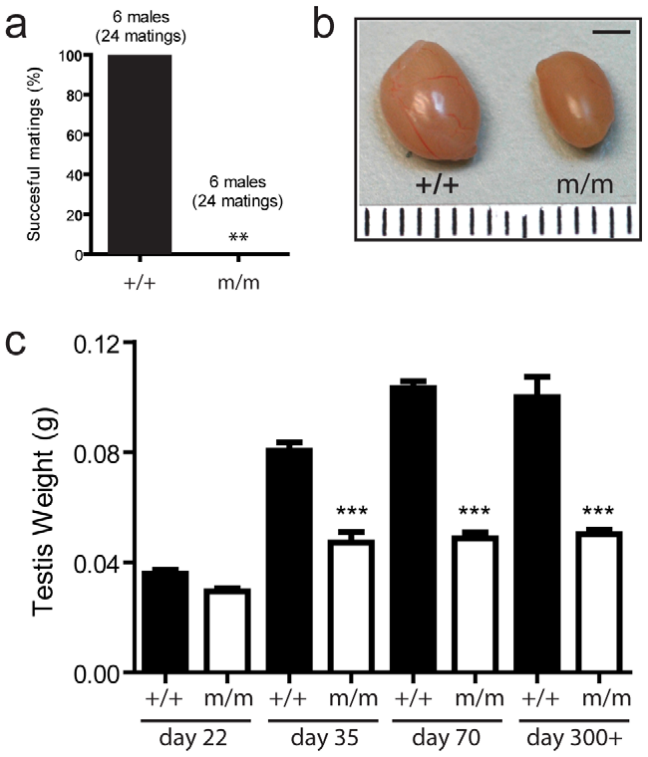
A novel mouse model of male-specific infertility. A screen for ENU-induced recessive mutations that cause male-specific infertility was undertaken. (a) In one pedigree (PED-JP5), six infertile males were identified through matings to a total of four or more CD1 females each. A significant reduction in testis weight in mutant animals was noted (b, c), which was first observed at day 35 and remained consistently 60% of wild-type weight when compared at both day 70 (adulthood) and at around one year of age (days 300–365) (c). (+/+ = Wild-type, m/m = recessive mutant, bar = 2 mm, ** p = 0.0022).

To identify novel genes important for the promotion of male fertility, a screen for recessive mutations that cause male-specific infertility was undertaken as part of a screen for developmental phenotypes (for details see Materials and Methods). In one pedigree (PED-JP5), two G2 females out of the four tested gave rise to a total of six infertile males, confirmed through consecutive matings to a total of four or more CD1 females per male (Figure 1a). Subsequent pedigree analysis showed that the infertility trait had an autosomal-recessive mode of inheritance with complete penetrance and expressivity in homozygous mutant males (m/m). Conversely, heterozygous males and females of all genotypes were fertile (data not shown).

### Infertile males have reduced testis weight

Infertile males were culled and examined at various ages to establish the phenotypic changes underlying the infertility trait. Infertile males displayed no significant difference in bodyweight, or ano-genital distance (AGD) when compared to WT littermates at post-natal days (day) 22, 35, and 70 (n = 5–18 per group, data not shown). However, a significant reduction in testis weight in mutant animals was noted (Figure 1b, 1c), which was first observed at day 35 and remained consistently 60% of wild-type weight when compared either in early adulthood or later (approximately one year) (p<0.001) (Figure 1c). Gross inspection revealed no other obvious phenotypic difference.

### Genetic mapping identifies *Katnal1* as the causal gene

**Figure 2:**
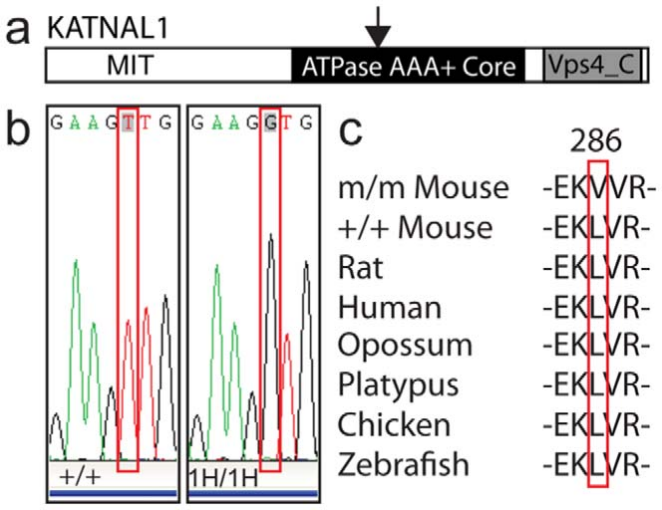
Genetic mapping identified a loss of function allele of *Katnal1* as the causal genetic lesion. Genome-wide SNP linkage analysis identified a single region of distal chromosome 5 as the site of the causal mutation. Quantitative RT-PCR analysis and DNA sequencing analysis of genes within the critical region identified a single point mutational change (Thymine to Guanine) within exon seven of the gene encoding the putative microtubule severing protein Katnal1 (a, b) (The mutated allele was designated *Katnal1^1H^*). The point mutation within *Katnal1^1H^* generates a Leucine to Valine substitution at residue 286, within the conserved ATPase AAA-Core domain of the KATNAL1 protein (a, b). (c) Comparative genome analysis of KATNAL1 peptide sequence across diverse species demonstrated complete conservation of the Leucine residue for at least 400 million years.

To identify the genetic lesion responsible for the observed reduction in testis weight and infertility, a genome-wide SNP linkage analysis was employed to identify homozygous regions of DNA derived from the initial ENU-treated C57BL/6J founder, in ten infertile G3 males. This highlighted a single genomic locus, covering 2 Mbp of distal chromosome 5 (between SNP rs6349247 and SNP rs13478592) common to all mutant males, which contained 17 candidate genes. To identify the causal gene, both gene expression analysis and DNA sequencing approaches were employed. Quantitative RT-PCR analysis of every splice-variant transcript from all 17 genes from WT, heterozygous and homozygous day 22 testes (onset of puberty) revealed no significant gene expression changes coincident with the infertility phenotype (n = 4–5 per genotype, data not shown). However, DNA sequencing of every exon from all 17 candidate genes within the critical region in both fertile and infertile males did identify a single homozygous point mutational change (Thymine to Guanine) within exon seven of the gene encoding the novel microtubule severing protein KATNAL1 (Figure 2a, 2b). This novel allele was designated *Katnal1^1H^*. The mutation in *Katnal1^1H^* serendipitously generated an HpyAV restriction-enzyme recognition site which was exploited for genotyping purposes (data not shown). This assay was predictive of phenotype with 100% accuracy in all animals tested (n = 186).

### Comparative genome analysis suggests the mutation in *Katnal1* is functionally significant

The point mutation within *Katnal1^1H^* generates a Leucine to Valine substitution at residue 286, within the conserved ATPase AAA-Core domain of the KATNAL1 protein (Figure 2a). Because this substitution appeared relatively conservative (both Leucine and Valine are branch-chained, hydrophobic amino acids), we undertook comparative genome analysis of KATNAL1 peptide sequence across diverse species (www.ensembl.org) with a view to establishing the significance of a Leucine residue at this position. The Leucine residue at this position in KATNAL1 was conserved in all species examined, suggesting that this residue has been under strong selection pressure for >400 million years (Figure 2c).

### KATNAL1 is a microtubule severing protein

**Figure 3:**
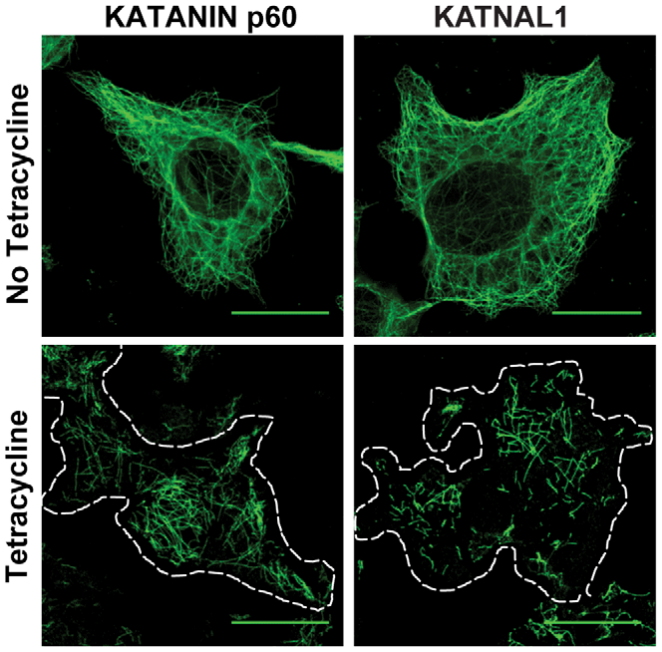
KATNAL1 is a microtubule severing protein. Hek293 cells were stably transfected with constructs encoding either KATANIN p60 or KATNAL1 protein. Separate wells were treated +/− tetracycline or vehicle for a period of 12 hours, fixed then stained for α-tubulin as a marker of microtubules. This demonstrated that, like KATANIN p60, KATNAL1 functions as a microtubule severing protein. Scale bars = 20 µm.

To establish whether KATNAL1 had similar function to KATANIN p60, HEK293T cells were stably transfected with cDNA for either *Katanin p60* or *Katnal1*, and protein expression induced using a tetracycline-inducible promoter. Addition of tetracycline resulted in disruption of the cellular microtubule lattice in both *Katanin p60* and *Katnal1* transfected cells within 12 hours confirming KATNAL1 functions as a microtubule severing protein (Figure 3).

### *In vitro* functional analysis confirms the mutation in *Katnal1* is functionally significant

**Figure 4:**
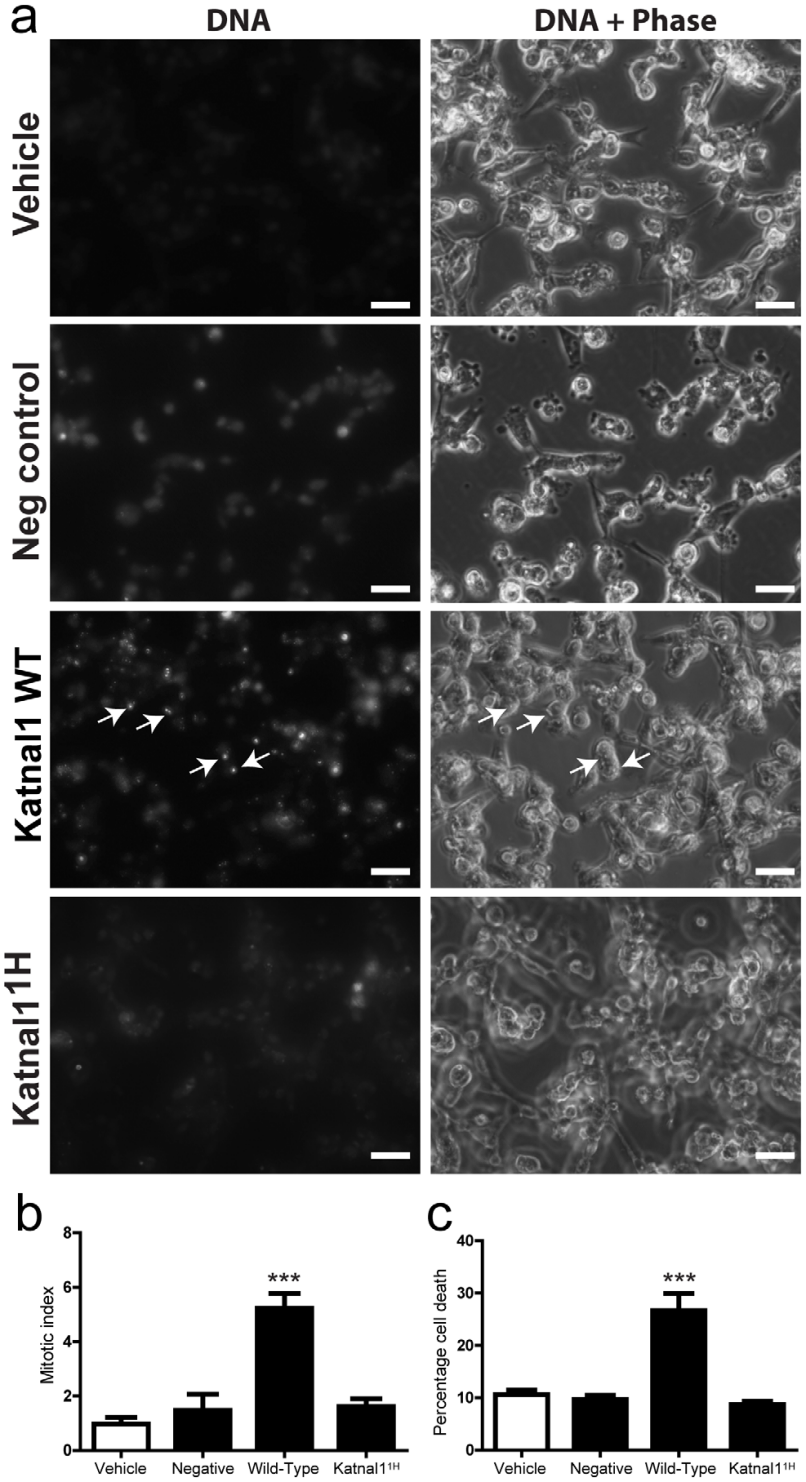
The identified mutation in *Katnal1^1H^* results in loss of function. (a, b) Transduction of HT1080 cells with lentiviral particles containing the WT cDNA of *Katnal1* under the control of the CMV promoter results in a significant increase in numbers of cells arrested in mitosis (arrows) 48 hours post-transduction. Conversely, numbers of cells in mitotic arrest does not differ from controls following transduction of the mutant *Katnal1^1H^* cDNA. (c) The arrest of cells during mitosis is followed by a significant increase in cell death in cells transduced with WT but not mutated *Katnal1^1H^*. Together these data confirm that the observed mutation in *Katnal1^1H^* generates a loss of function allele. Bar = 50 µm (*** = p<0.001).

To empirically establish that this mutation has functional significance, we generated Lentiviral vectors expressing either the WT or *Katnal1^1H^* cDNA under the control of the strong CMV promoter and transduced cells *in vitro*. Having shown that induced-expression of a functional KATNAL1 protein increases microtubule severing, we hypothesised that disruption to microtubule dynamics through over-expression of functional KATNAL1 would lead to cellular arrest during mitosis, followed by cell death. Indeed, over-expression of the WT KATNAL1 protein in HT1080 cells significantly increased both the number of cells in mitosis (p<0.0001) and significantly increased overall cell death compared to vehicle or control lentivirus when tested at 48 hours post-infection (p<0.001), whereas over-expression of mutant *Katnal1^1H^* cDNA failed to induce any increase in number of cells in mitosis or any change in overall cell death relative to controls (Figure 4). We therefore deduced that the observed male infertility phenotype in these mice resulted from a single base-pair change encoding a conserved residue of KATNAL1, and that *Katnal1^1H^* is a recessive loss-of-function allele.

### *Katnal1* is expressed in multiple tissues including the testis

**Figure 5:**
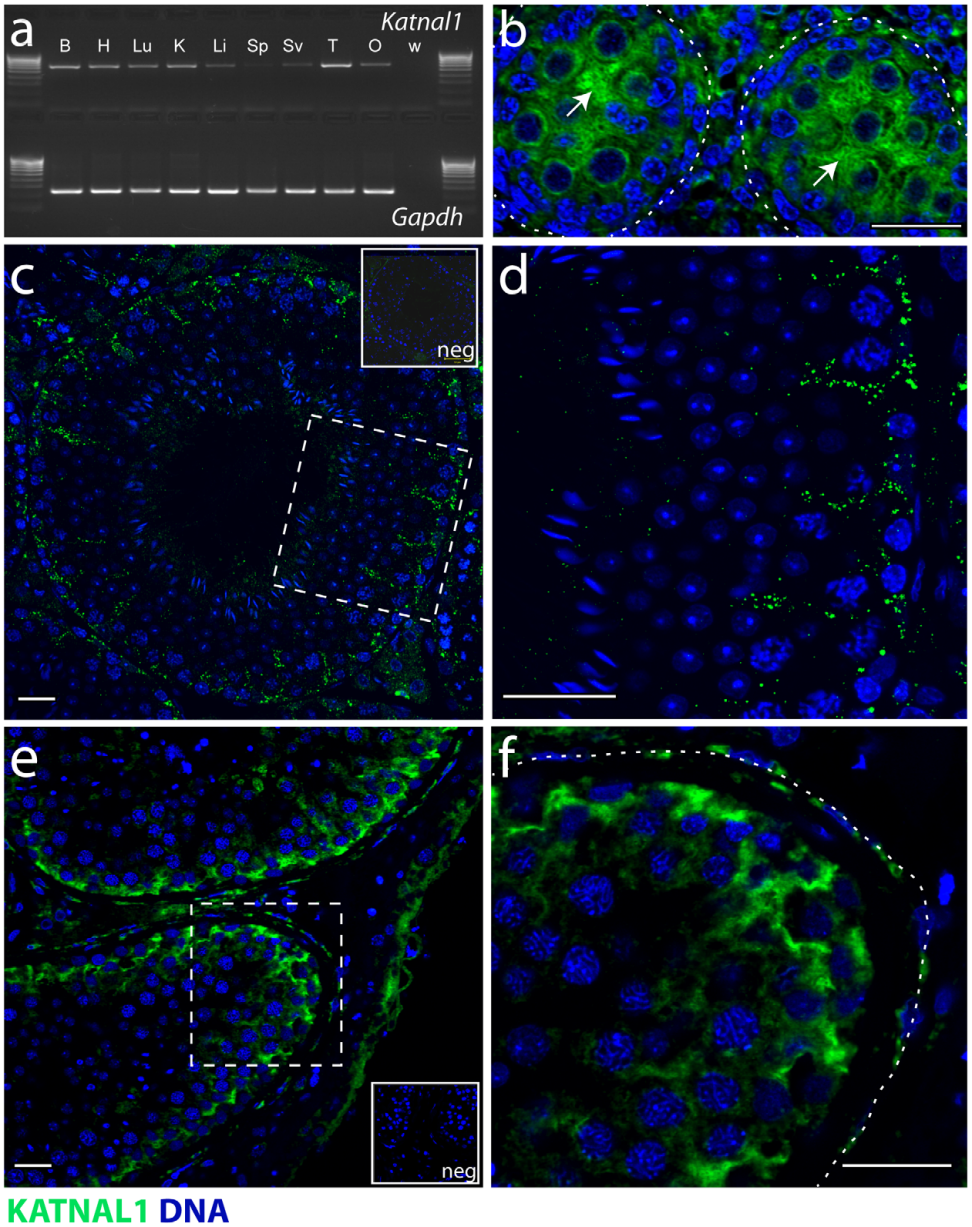
Katnal1 is widely expressed. Testicular Katnal1 expression is detected from 15.5 dpc and is SC-specific within the seminiferous epithelium throughout life. (a) RT-PCR analysis identified Katnal1 gene expression in a wide panel of tissues including the testis. To establish the cellular localisation of KATNAL1, a custom antibody was designed to specifically recognise KATNAL1. Using this antibody, an immunohistochemical time-course analysis confirmed testicular Sertoli cell (arrowed) expression of KATNAL1 from 15.5dpc onwards (b), and that KATNAL1 protein is restricted to Sertoli cells of the seminiferous epithelium throughout postnatal life where it is localised at a number of discrete foci (c) (dotted box enlarged in (d)). Immunohistochemical interrogation of adult human testis sections using the KATNAL1 antibody (e) (dotted box enlarged in (f)), confirmed a similar localization for KATNAL1 in both human and mouse testis. (B = Brain, H = Heart, Lu = Lung, K = Kidney, Li = Liver, Sp = Spleen, Sv = Seminal Vesicle, T = Testis, O = Ovary, W = water control. Bars in (c, d, e and f) = 20 µm; Neg = peptide blocked control).

Given that the loss of function of KATNAL1 resulted in a testicular phenotype, we next conducted RT-PCR analysis, interrogating from exon eight to the 3′-untranslated region of *Katnal1* (which amplifies both published splice variants that contain the functional ATPase AAA Core domain, www.ensembl.org) on RNA taken from a panel of tissues of adult C57BL/6J mice, to establish the body-wide expression pattern of *Katnal1*. This analysis demonstrated that *Katnal1* is widely expressed, with gene expression detectable in all tissues examined including brain, heart, lung, kidney, liver, spleen, seminal vesicles, and ovary, in addition to the predicted testicular expression (Figure 5a).

### Testicular *Katnal1* expression is detected from 15.5 dpc and is SC–specific throughout life

Having identified *Katnal1* expression in the testis, an immunohistochemical time-course analysis of testis sections from several pre- and post-natal ages was undertaken using a custom polyclonal antibody designed to specifically detect KATNAL1 (Figure S1). This confirmed expression of KATNAL1 from 15.5dpc onwards (Figure 5b) and that KATNAL1 protein is restricted to Sertoli cells within the seminiferous epithelium throughout postnatal life (Figure 5c, 5d and data not shown). In post-natal life, KATNAL1 protein was observed throughout the Sertoli cell cytoplasm, with apparent concentration in a number of discrete foci, (Figure 5c, 5d). Immunohistochemical interrogation of adult human testis sections (n = 3) using the KATNAL1 antibody confirmed a similar expression pattern for KATNAL1 in both human and mouse testis (Figure 5e, 5f).

### Reduction in testis weight is the result of a reduction in numbers of post-meiotic germ cells

Once we had unequivocally established that KATNAL1 function had been ablated in this model, and localised the primary cellular site of disruption as the SC, comparative analyses against wild-type littermates were undertaken at three key ages spanning the first, and subsequent waves of spermatogenesis (days 22, 35 and 70), to establish the consequences of loss of KATNAL1 upon testicular function.

**Figure 6:**
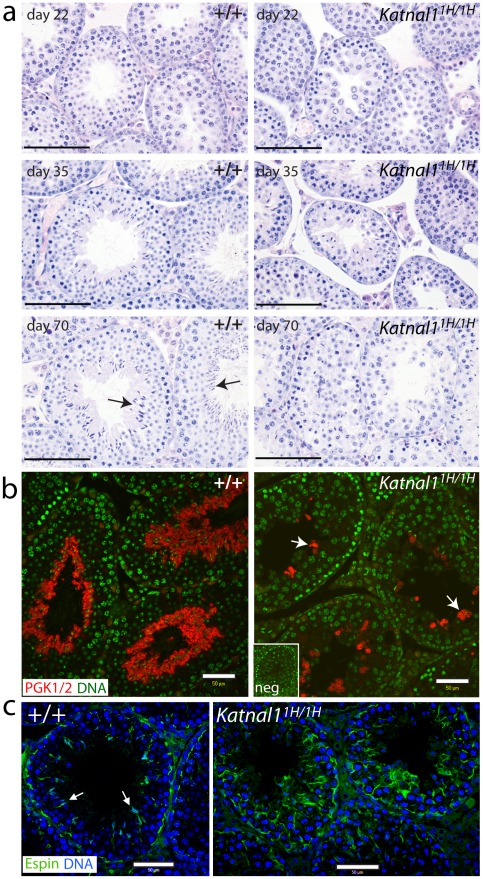
Histological analysis of testis sections reveals disruption to the seminiferous epithelium associated with a reduction in numbers of post-meiotic germ cells. (a) Testes from wild-type and *Katnal1^1H/1H^* animals appear similar at day 22 when examined by light microscopy. However, at day 35 disruption to the seminiferous epithelium is apparent in mutant testes, consistent with a significant reduction in seminiferous tubule diameter. At day 70, the seminiferous epithelium in the *Katnal1^1H/1H^* testis appears disorganised and elongate spermatids (arrowed in WT) are almost absent. (b) The apparent reduction in elongate spermatid numbers was confirmed (arrows) using an antibody against PGK1/2 which marks post-meiotic germ cells from step 8. (c) The reduction in elongate spermatids was associated with a clear reduction in apical ectoplasmic specialisations (marked by Espin) in *Katnal1^1H/1H^* testes (arrows in +/+). (+/+ = Wild-type; neg = no-primary control; Bar = 100 µm unless stated).

At day 22 (early puberty) examination of testicular paraffin-sections revealed no gross histological differences between WT and homozygous null animals (Figure 6), although a reduction in germ cells was apparent by stereological analysis (see below). However, at day 35 (mid-puberty), clear histological differences in the seminiferous epithelium were apparent (Figure 6), including a significant reduction in seminiferous tubule diameter (p<0.001, n = 5, data not shown). At day 70, the seminiferous epithelium was severely disrupted with evidence of SC vacuolation and an apparent reduction in numbers of elongate spermatids (figure 6a). Quantification of SC and GC numbers at all three ages revealed no significant difference either in numbers of SCs or numbers of spermatogonia or spermatocytes at any age (Table 1). However, at day 22, day 35 and day 70, a significant reduction in numbers of spermatids was observed (Table 1). This reduction was confirmed histologically using antibodies against PGK1/2 which specifically marks spermatids from step eight onwards [25] (Figure 6b), and Espin, which marks the ectoplasmic specialisation junctional complex between SCs and spermatids (Figure 6c).

**Table 1:** Sertoli Cell and Germ Cell composition of the testes.

		Average volume (mm^3^/testis)				
Age	Genotype	Sertoli	Spermatogonia	Spermatocyte	Round Spermatid	Elongate Spermatid
22	+/+	0.89±0.10	0.50±0.04	5.73±0.37	1.56±0.20	NA
22	1H/1H	0.97±0.08	0.57±0.05	5.98±0.40	1.07±0.15 (69%) *	NA
35	+/+	1.37±0.13	0.85±0.09	8.65±0.96	6.59±0.78	3.56±0.75
35	1H/1H	1.44±0.16	0.80±0.06	6.89±0.72	2.42±0.50 (37%) ***	1.00±0.20 (27%) **
70	+/+	1.14±0.15	0.73±0.14	6.60±0.87	5.50±1.07	4.01±0.87
70	1H/1H	0.87±0.13	0.52±0.09	5.08±0.66	1.53±0.17 (28%) **	0.59±0.05 (15%) **

Comparison of Sertoli and Germ cell composition of the testes from Wild-type (+/+) and homozygous *Katnal1^1H/1H^* (1H/1H) mutant mice at d22, d35 and d70. (n = 5 per group; Mean ± SEM).

### Reduction in numbers of post-meiotic germ cells is the result of premature exfoliation from the seminiferous epithelium

**Figure 7:**
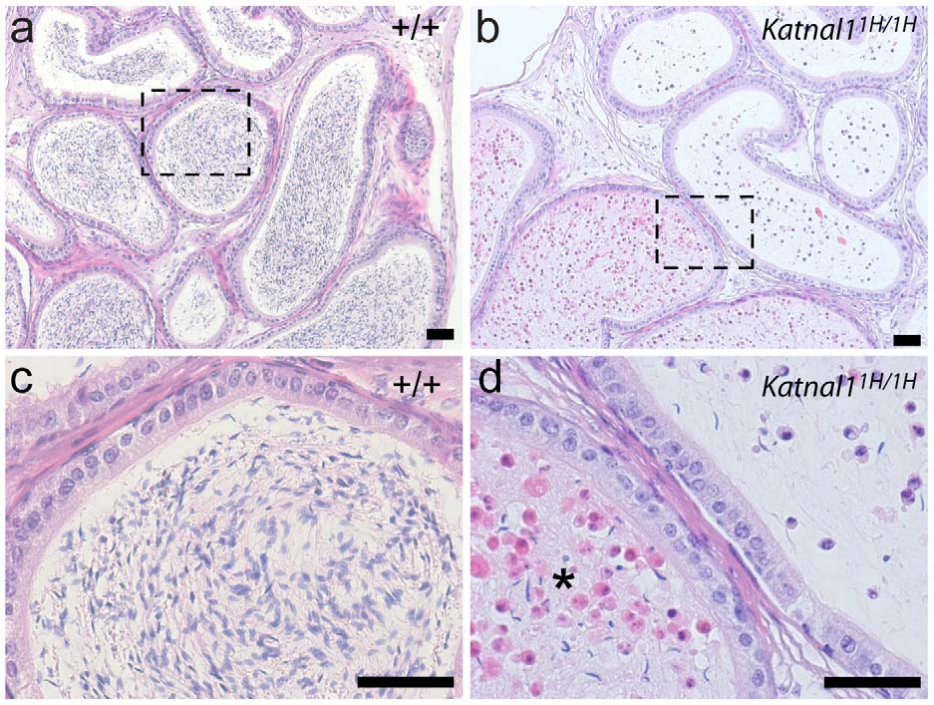
Reduction in post-meiotic germ cells is due to premature sloughing from the seminiferous epithelium. Sections of epididymides from homozygous mutant and WT littermates were examined. In WT animals the epididymal lumen was filled with spermatozoa throughout the length of the epididymis (a, - boxed area enlarged in c). However, in homozygous *Katnal1^1H/1H^* littermates, recognisable spermatozoa were almost absent, instead the caput and corpa epididymides contained immature, non-condensed, germ cells (b), which collected as a plug of proteinaceous cellular debris (*) in the cauda epididymis (b, boxed area enlarged in d). Bars = 20 µm.

To establish whether the significant reduction in spermatid number was the result of a reduction in production of spermatids, an increase in spermatid apoptosis, or the result of exfoliation of developing spermatids from the seminiferous epithelium, testes and epididymides from homozygous mutants and WT littermates were examined. Colorimetric immunohistochemistry using an antibody against the apoptotic marker Cleaved-Caspase-3 identified no difference in numbers of apoptotic germ cells in testis sections from mutant versus WT animals (n = 5, data not shown), thus increased spermatid apoptosis could not explain the observed phenotype. In WT animals the epididymal lumen was, as expected, filled with spermatozoa throughout its length (Figure 7a, 7c). However, in *Katnal1^1H/1H^* littermates, recognisable spermatozoa were much reduced, and instead the caput and corpus epididymides contained exfoliated immature germ cells (Figure 7b) which collected as a plug of proteinaceous cellular debris in the cauda epididymis (Figure 7d). Together these data demonstrated that the primary phenotypic defect in *Katnal1^1H/1H^* mutants is premature exfoliation of spermatids form the seminiferous epithelium.

### KATNAL1 co-localises with SC microtubules but is restricted to basal regions in mutant testes

**Figure 8:**
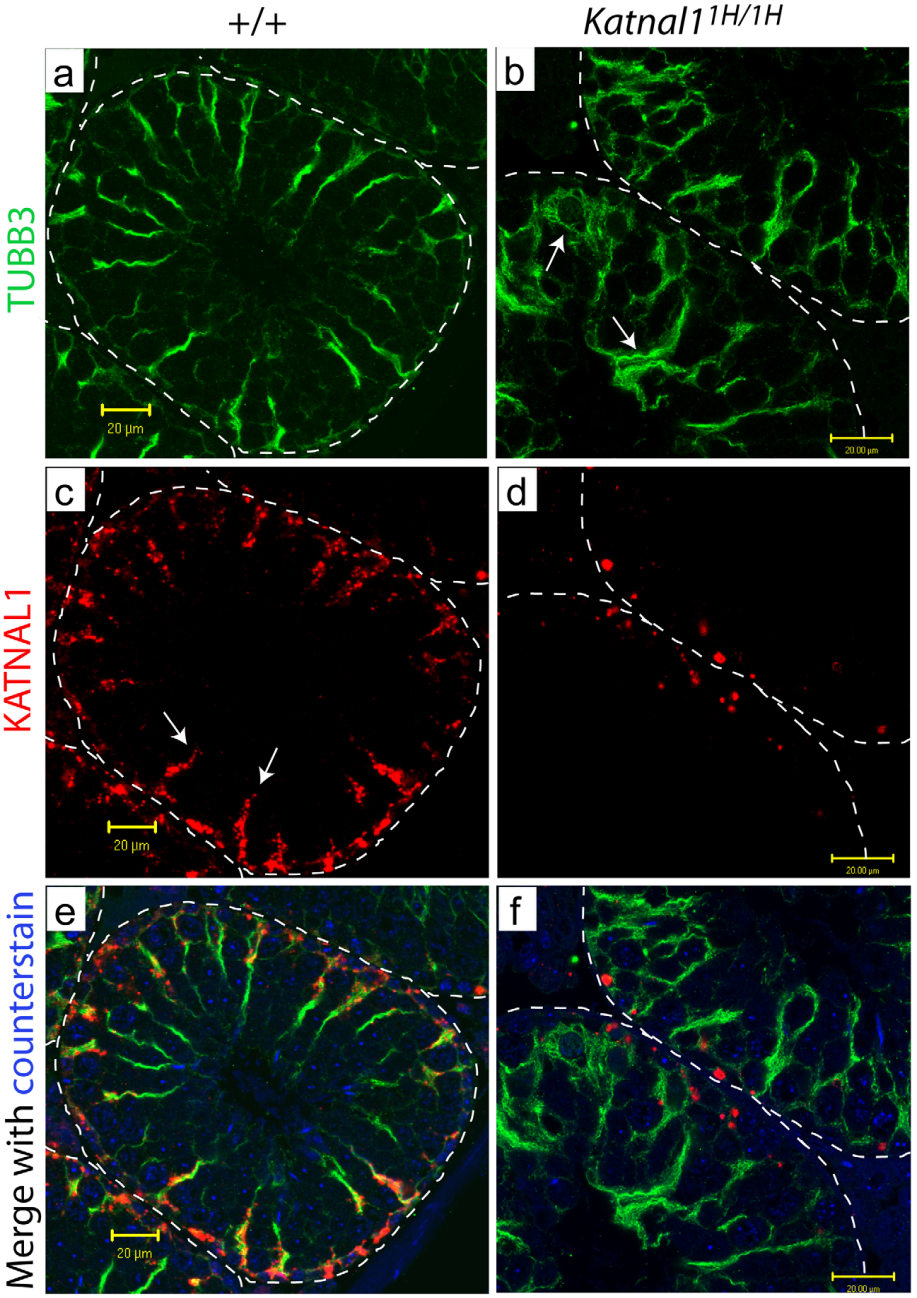
Katnal1 co-localises with Sertoli cell microtubules, but is restricted to basal regions in mutant testes. At d35, immunohistochemical localisation of the Sertoli cell-specific isoform of beta-tubulin TUBB3 reveals an apparent disruption to the microtubule network in *Katnal1^1H/1H^* testes, (a, b). In Wild-Type animals, KATNAL1 localisation (arrows) tracks the SC microtubule network from basal to apical regions (c, e). Conversely, the mutant KATNAL1 protein is restricted to the basal region of Sertoli cells in *Katnal1^1H/1H^* animals (d, f). Images representative of stage VI of the spermatogenic cycle. Bars = 20 µm.

To establish the mechanism underlying the premature exfoliation of spermatids in the *Katnal1* mutants, we examined colocalisation of KATNAL1 with a Sertoli cell-specific isoform of beta-tubulin TUBB3 [26]. Localisation of TUBB3 revealed an apparent disruption to the microtubule network in mutant testes compared to WT (Figure 8a, 8b). In testes from WT animals, KATNAL1 co-localises with SC microtubules throughout the basal and adluminal compartments of the SCs (Figure 8a, 8c, 8e), however, in *Katnal1^1H/1H^* animals, the mutated KATNAL1 protein was restricted to the basal compartment of SCs (figure 8d, 8f).

### SCs from *Katnal1^1H/1H^* animals contain reduced numbers of stable microtubules

**Figure 9:**
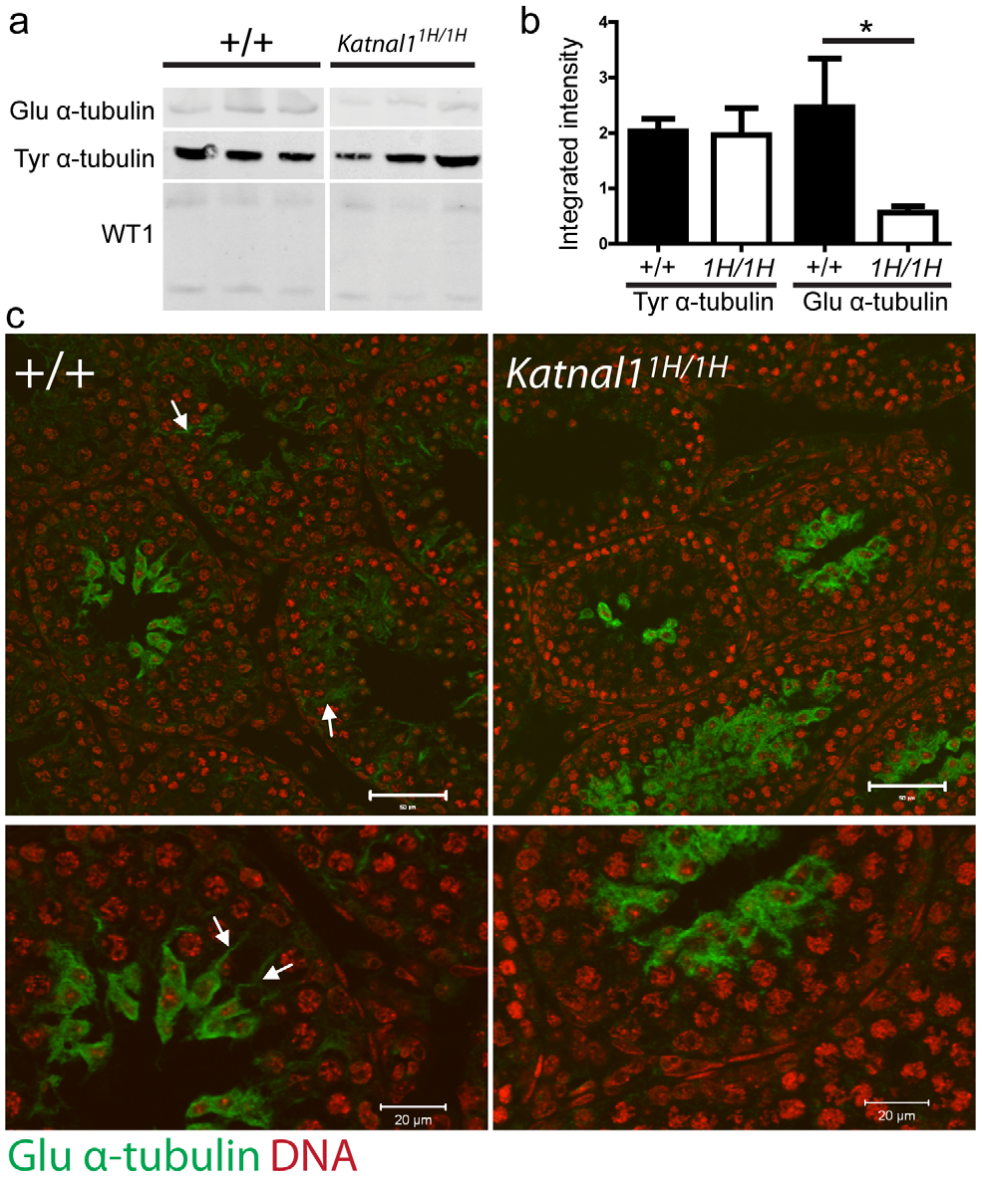
Loss of KATNAL1 function leads to reduced numbers of stable microtubules in SCs. (a) Western blot analysis and (b) quantification of stable (glu) and dynamic (tyr) α-tubulin on d22 testes from wild-type and homozygous mutant animals reveals a significant reduction in numbers of stable microtubules in testes from *Katnal1^1H/1H^* mutants (n = 5–7). (c) Immunohistochemical localisation of glu-α-tubulin on corresponding d22 testis sections localises stable microtubules to SC cytoplasm (arrows) and developing spermatids and in Wild-type animals. Conversely stable microtubules are below detection limits in much of the SC cytoplasm in homozygous mutant testes. (WT1 = Wilms Tumour 1, SC-specific loading control).

The tubulin tyrosination cycle involves the cyclical removal and re-addition of the carboxy-terminal tyrosine residue of the α-tubulin [27]. Removal of the Tyrosine residue exposes the carboxy-terminal Glutamic acid. As a consequence of the kinetics of this system, tyrosinated (tyr)-tubulin is the main component of dynamic microtubules, whereas glutamic acid (glu)-tubulin is a marker of long-lived stable microtubules [28]. Because antibodies against glu-tubulin bind only the exposed carboxy-terminal, glu-tubulin provides an excellent surrogate marker for changes in numbers of stable microtubules. To examine the impact of loss of function of KATNAL1 on SC microtubule dynamics quantitative (Western blot) and spatial (immunohistochemical) analysis of dynamic and stable microtubules was undertaken at d22. Whilst tyr-tubulin microtubules were unaffected in testes from *Katnal1^1H/1H^* animals, there was a statistically significant reduction in glu-tubulin in *Katnal1^1H/1H^* testes (Figure 9a, 9b). Subsequent immunohistochemical localisation of glu-tubulin on d22 testis sections identified staining of similar intensity associated with developing spermatids in both WT and *Katnal1^1H/1H^* animals (Figure 9c), suggesting that, at least in part, the observed reduction in glu-tubulin was likely associated with the reduction in total spermatid numbers in d22 *Katnal1^1H/1H^* testes (Table 1). However, in addition to spermatids, glu-tubulin was also widely detected throughout SCs in wild-type testes, yet fell below detection limits throughout the majority of cytoplasm of the SCs from *Katnal1^1H/1H^* animals (Figure 9c).

## Discussion

Utilising random ENU mutagenesis, we have identified a new mouse line displaying male-specific infertility due to a point mutation in the highly conserved ATPase domain of the novel microtubule severing protein KATNAL1. We demonstrate that *Katnal1* is expressed in testicular Sertoli cells from 15.5 dpc onwards, co-localises with SC microtubules *in vivo*, and that loss of function leads to male-specific infertility through disruption of microtubule dynamics and premature exfoliation of post-meiotic germ cells from the seminiferous epithelium. This is the first description of an *in vivo* function for this novel microtubule severing protein, a finding that has implications for our understanding of SC microtubule dynamics, the promotion of male fertility, and as a potential target for male contraceptive development.

The use of the chemical mutagen ENU to produce novel mouse models of male infertility is now well established [29], [30]. This approach has several advantages, not least as it requires no *a priori* information about the causal gene, which can be identified by a relatively straightforward combination of positional cloning and DNA sequencing. We utilised this approach to identify a mouse line displaying male-specific infertility, associated with a significant reduction in testis weight. However, the reduction in testis weight remained consistent through to approximately one year of age suggesting that the genetic lesion did not result in cumulative degeneration with aging, and thus that the infertility phenotype was likely to be due to repeated failure at a specific aspect of spermatogenesis.

Using both classical and molecular genetic analysis we identified *Katnal1* as the gene responsible for the infertility phenotype. KATNAL1 derives from the same gene family as KATANIN p60 (66% identity, 78% conserved), a well described microtubule severing protein important in neuronal plasticity [18]. Functional katanin complexes are assembled from two distinct gene products, KATANIN p60 and KATANIN p80, which oligomerise to form homo-hexamers before combining to produce a functional protein [31]. The KATANIN p60 peptide has three functional domains, an N-terminal Microtubule Interacting and Trafficking (MIT) domain [22], an AAA ATPase domain [32], and a C-Terminal Vsp4_C domain, important in oligomerisation [32]. Interpro analysis (www.ebi.ac.uk/interpro) of KATNAL1 identified both the AAA ATPase domain and the Vsp4_C domain, based upon sequence homology, but the N-terminal primary sequence of KATNAL1 is less conserved when compared to KATANIN p60 suggesting KATNAL1 has a related, yet distinct function from KATANIN p60. *Ab intitio* structure modelling using ROSETTA confirmed the presence of a Microtubule Interacting and Transport (MIT) domain at the N-terminal of KATNAL1 [22], We (this study), and recently others [23], have now unequivocally confirmed that KATNAL1 functions as a microtubule severing protein.

Whilst the mutation in the *Katnal1^1H^* allele results in a relatively conservative amino acid substitution (Leucine-Valine), comparative analysis across diverse species demonstrates the significance of the Leucine residue at this position, with complete conservation across 400 million years of evolution. The mutation is localised to the ATPase Core domain of the KATNAL1 peptide. Hydrolysis of ATP is known to be essential for microtubule severing function of the related KATANIN p60 [18], suggesting that the mutation in *Katnal1^1H^* may result in a disruption to ATP hydrolysis function in KATNAL1. This requires further investigation. Nevertheless, both the recessive nature of the phenotype and our examination of protein function through *in vitro* over-expression studies support a ‘loss-of-function’ (LOF) model for the mutated *Katnal1^1H^* allele.

KATNAL1 is widely expressed throughout many body systems. It is perhaps surprising therefore that no obvious phenotypic difference outside of the testis was apparent in *Katnal1^1H/1H^* animals suggesting a degree of functional redundancy exists to support LOF of KATNAL1 in other body systems. Given the shared characteristics of SCs and neurones [4], and the extensive work on the related proteins KATANIN p60 [16], [17], [20], [33]–[35] and SPASTIN [36]–[39] in neurones, a further, detailed investigation, focused upon potential behavioural or neuronal endpoints in the *Katnal1^1H/1H^* mutants is an important next step.

Within the testis, KATNAL1 is exclusively expressed in SCs in human and in mouse from 15.5dpc. KATANIN p60 has previously been implicated in both mitosis and female meiosis where it functions to increase the pool of short microtubules required for chromosome separation [40]–[43]. As KATNAL1 protein is present in SCs from prenatal life, we could hypothesise a similar role for KATNAL1 during mitotic division of SCs. However, we observed no difference in SC number at any age in the *Katnal1^1H/1H^* mutant, suggesting KATNAL1 is dispensable for completion of SC mitosis. The role for KATNAL1 in SCs prior to puberty therefore remains to be established.

Further to this, no reduction in numbers of pre-meiotic germ cells was observed at any age suggesting the systems to functionally and nutritionally support spermatogonia and spermatocytes were not affected by loss of KATNAL1. Furthermore, the phenotype does not appear to be degenerative or cumulative over time, with SCs continuing to support the pre-meiotic germ cells throughout life suggesting that the phenotype represents repeated failure coincident with a specific stage of germ cell development. With no evidence of increased germ cell apoptosis in the *Katnal1^1H/1H^* mutant, and with epididymides filled with immature germ cells, we can conclude that KATNAL1 function is essential for retention of spermatids during spermiogenesis, and that fundamental failure at this specific stage of spermatogenesis is the primary cause of the observed infertility phenotype.

KATNAL1 is not uniformly distributed throughout the SC cytoplasm, but is localised in discrete foci localised throughout the basal and adluminal regions. In wild-type animals, KATNAL1 co-localises with SC microtubules, however, in *Katnal1^1H/1H^* animals, mutant KATNAL1 is restricted to the basal region of SCs. Whether this mis-localisation is the direct result of the mutation, or a consequence of other changes in SC function such as changes in cell polarity brought about by the LOF of KATNAL1 requires further investigation.

Microtubules play several important roles within Sertoli cells during spermatogenesis, including maintaining SC architecture, facilitate the secretion of tubule fluid, intracellular vesicle transport and the translocation of spermatids during maturation, each of which are reliant upon dynamics changes in microtubules during the differing stages of the spermatogenic cycle (for review see [7]). Severing not only promotes microtubule destruction, but is also important for the creation of new microtubules, as severing of longer microtubules generates “seeds” for further microtubule polymerization [44], [45]. *Katnal1^1H/1H^* animals display a reduction in numbers of stable microtubules with SCs, suggesting KATNAL1 function is required in SCs to seed development of stable microtubules. This reduction in numbers of stable microtubules is likely to be a key factor in the premature exfoliation of immature spermatids from the seminiferous epithelium, perhaps simply because the numbers of microtubules attached to each spermatid are insufficient in numbers to ensure the spermatid remains attached, the transport of nutrients to the developing spermatids is inadequate, or perturbation of microtubule dynamics interferes with correct development of the apical ectoplasmic specialisation – which were obviously reduced in *Katnal1^1H/1H^* testes. Future studies will focus upon detailing the molecular interactions between KATNAL1 and other factors promoting correct spermiogenesis.

In conclusion, identification of KATNAL1 as an essential promoter of male fertility and functional analysis of its role within testicular SC represents an important first step towards understanding the molecular mechanisms underlying SC microtubule remodelling. Such information will be of utility both for increasing our understanding of male infertility and the development of treatments and non-hormonal male contraceptives.

## Materials and Methods

### Ethics statement

All mice were bred under standard conditions of care and use under licence approval from the UK Home Office. Appropriate ethical approval was obtained for use of human testis tissue (Approval number: 2006-588-fs; Professor S. Kliesch and Professor S. Schlatt, Centre of Reproductive Medicine and Andrology (CeRA), Münster, Germany).

### Generation of infertile males

As part of a screen for developmental mutants a recessive screen for male fertility was undertaken. C57BL/6J males were treated with 3×100 mg/kg ENU, with each dose a week apart, to induce random mutations, in spermatogonial stem cells. Two months after completion of treatment the males were outcrossed to C3H/HeH females. Only outcrosses that yielded first litters after twelve weeks from the end of treatment were used to ensure that the ENU treatment had been successful. G1 males arising from the outcross were used to found pedigrees by crossing to C3H/HeH females to generate G2 females. The G2 females were backcrossed to their G1 father to produce G3 males. If the G2 female carried a recessive mutation then the probability of homozygosity in a G3 male would be 0.25. From each of at least four G2 females, four or more G3 males were screened for reduced fertility by mating each to single CD1 females. The CD1 females were culled at 15.5 dpc and embryos counted. All males with greater than ten offspring on opening at 15.5 dpc were classified as Wild-Type (WT) (CD1 females have a mean first litter size of 13.5 on crossing to WT males, data not shown). If there were less than ten fetuses the male was retested with second CD1 female. Six G3 males derived from two G2 females in one pedigree, PED-JP/5, produced no fetuses at all. Each of these putatively sterile males was further bred sequentially to four or more CD1 females. None of these further matings produced fetuses. Comparable breeding of six male litter-mates resulted in 100% successful matings, defined as production of greater than 10 fetuses per female.

### Recovery of tissue

Mice were culled at various pre- and postnatal ages and tissues were collected, weighed and snap-frozen or fixed in Bouins fixative as previously described [46]. Bouins fixed tissue was processed and embedded in paraffin wax and 5 µm sections cut for histological analysis and stained as previously described [47].

### Genetic mapping

Sixty-three SNPs polymorphic between C57BL/6J and C3H/HeH providing coverage of 2–5 SNPs per chromosome were interrogated in DNA from ten infertile males using Pyrosequencing on a PSQ HS 96, according to manufacturer's instructions (Qiagen, Crawley UK). A single shared C57BL/6J haplotype was localised in all ten males to distal chromosome 5. Addition of five further SNPs in this region resolved a minimal critical region between SNP rs6349247 and SNP rs13478592.

### RT–PCR

Testicular RNA was isolated from day 22 WT, heterozygous and homozygous mice (n = 4–5 per group) as previously described [46]. cDNA was prepared using the SuperScript® VILO™ cDNA Synthesis Kit (Invitrogen, Paisley, UK) according to manufacturer's instructions. Quantitative PCR analysis was performed on testes from day 22 WT, heterozygous and homozygous mice as previously described [46]. The expression of each gene was related to Luciferase mRNA, added to each sample prior to RNA isolation as an external control, and all genes were expressed per testis. For Non-quantitative RT-PCR analysis of *Katnal1* and *Gapdh* expression, cDNA was amplified using Biomix red according to manufacturer's instructions (Bioline, London, UK), and intron-spanning primers GCCCCTACCACGATCTTCA and CCCAGAGATGGAAGAGCTGA (Katnal1) and CTGCACCACCAACTGCTTAGC and ATGCCAGTGAGCTTCCGTTC (GAPDH) and resolved on a one percent agarose gel against Hyper Ladder IV (Bioline).

### DNA sequencing

Every published exon (www.ensembl.org) from all 17 genes between SNP rs6349247 and SNP rs13478592 was amplified by PCR from genomic DNA collected from fertile and infertile males using Biomix Red (Bioline) according to manufacturer's instructions. Samples were purified using the High Pure PCR Product Purification Kit (Roche Diagnostics, Burgess Hill, UK). DNA sequencing was carried out on an ABI 3130xl Genetic Analyser (Applied Biosystems, Foster City, USA), as per manufacturer's instructions, and sequence traces analysed using Chromas Lite (www.technelysium.com.au).

### Genotyping

Mice were genotyped from ear or tail DNA. DNA spanning exon seven of *Katnal1* was amplified by PCR using primers TAAATGCACTGATCCCACCA and AAACTGCCAATCACCCTCAC and purified using the High Pure PCR Product Purification Kit (Roche Diagnostics). Amplicons were digested with the restriction enzyme HpyAV (New England Biosciences, Ipswich, USA) according to manufacturer's instructions and resolved on a one percent agarose gel against Hyper Ladder IV (Bioline).

### *In silico* analyses

Cross-species genomic comparisons and functional domain localisation within KATNAL1 were undertaken using the Ensembl database (www.ensembl.org).

### Site-directed mutagenesis

Full length WT *Katnal1* cDNA was purchased from Genecopeia (Rockville, USA), and the mutated allele generated using the GeneTailor™ Site-Directed Mutagenesis System (Invitrogen), according to manufacturer's instructions.

### Cell expression studies

Stably transfected HEK293 cells capable of expressing KATANIN p60 or KATNAL1 were generated using the Flp-In T-Rex Core Kit (Invitrogen, USA) as recommended by the manufacturer. cDNAs were amplified from total testis mRNA then cloned into Gateway compatible vectors as defined in the kit using the following primers *Katanin p60* GGGGACAAGTTTGTACAAAAAAGCAGGCTTCTTCACCATGAGTCTTCAAATGATTG and GGGGACCACTTTGTACAAGAAAGCTGGGTCCTAGCATGATCCAAACTC; and *Katnal1* GGGGACAAGTTTGTACAAAAAAGCAGGCTTCTTCACCATGAATTTGGCGGAGATTTGTGAG and GGGGACCACTTTGTACAAGAAAGCTGGGTCTCATGCAGACCCAAACTCAAC. Following antibiotic selection, cells were plated onto poly-L-lysine coated chamber slides. Katanin subunit expression was induced by the addition of 1 µg/ml tetracycline (Invitrogen, USA) for 12 hours then cells were fixed in 50∶50::acetone∶methanol for 20 min at room temperature. Microtubule structure was visualized by immunolabelling for α-tubulin (1 in 5000, T51168, Sigma Aldrich) followed by an Alexa Fluor 488 donkey anti-mouse secondary antibody (1 in 500, Invitrogen) in 1% BSA. Following extensive washing, microtubule length and distribution was analysed by confocal microscopy using a SP5 5-channel microscope (Leica Microsystems, Germany). Distribution was compared to identical cell preparations not subjected to tetracycline induction and to untransfected cells exposed to tetracycline. No differences were observed between control populations.

### Lentiviral generation

WT and mutant *Katnal1* cDNA were inserted upstream of an internal ribosome entry site fused upstream of humanised green fluorescent protein (IRES-hrGFP) using standard methods. Control IRES-GFP, WT *Katnal1*-IRES-GFP and Mutant *Katnal1*-IRES-GFP were amplified by PCR primers with *att*B1 and *att*B2r recognition sequences and shuttled using Gateway (Invitrogen) into pLenti6/V5-DEST vector. This was packaged to produce lentivirus as per manufacturer's instructions (Invitrogen). If required, lentivirus was further concentrated by centrifuging at 5000 rpm through a column with a 100,000 molecular weight cut-off. Viral titre was determined by serial dilution onto HT1080 cells with 6 µg/ml polybrene, 48 hours later green fluorescing cells were counted and converted into transduction units/ml (TU/ml).

### Lentiviral transduction and cell death assay

For Lentiviral transduction, HT1080 cells were passaged at 7500cells/ml and 100 µl aliquoted into duplicate wells of a black 96 well plates (Corning) containing Matrigel (Becton Dickinson Oxford, UK). On the day of transduction, media was removed, cells were washed in PBS and overlaid with 100 µl of optimem for 4 hours at 34°C. Four treatment groups were assigned: WT *Katnal1*-IRES-GFP; Mutant *Katnal1^1H^*-IRES-GFP; IRES-GFP; Optimem (as a vehicle control). Lentivirus was diluted in optimem to 1000 TU/ml and 100 µl added to the appropriate well for 6 hours at 34°C. Optimem was then replaced with complete media and cells cultured for 48 hours at 34°C. Cells were harvested using the MultiTox-Fluor Multiplex Cytotoxicity Assay (Promega, Southampton, UK) according to manufacturer's instructions, and assayed on an Optima FLUOstar (BMG Labtech, Offenburg Germany), according to manufacturer's instructions. Experiments were conducted in duplicate on six separate occasions.

### Mitotic index assay

HT1080 cells were cultured in DMEM (Gibco) containing 10% fetal calf serum at 37°C with 5%CO_2_. For Lentiviral transduction cells were plated at 200000 cells per well of a 6 well plate (Corning) containing Matrigel. On the day of transduction the media was removed from the cells and overlaid with media containing 6 ug/ml polybrene (Sigma) and Lentivirus to give an MOI of 10 when plated on the cells. Four treatment groups were assigned: WT *Katnal1*-IRES-GFP; Mutant *Katnal1^1H^*-IRES-GFP; IRES-GFP; DMEM + 10% FCS (as a vehicle control). Media was refreshed after 24 hours. After 48 hours cells were visualised and images captured using an Axiovert 200 inverted fluorescent microscope (Zeiss), the DNA was visualised by the addition of 5 µM DRAQ5 (Cell Signalling Technology, Danvers, MA) to the culture media.

### Western blotting

Protein was extracted from whole testis using 500 µl RIPA buffer (1% Triton X-100, 15 mM HEPES-NaOH (pH 7.5), 0.15 mM NaCl, 1% sodium deoxycholate, 0.1 sodium dodecyl sulphate (SDS), 1 mM sodium orthovanadate, 10 mM EDTA). 20 µg of protein was separated on a 4–12% Bis-Tris gel (Invitrogen), transferred to Immobilon –Fl membrane (Millipore) and probed with either rabbit α-Katnal1 (Covalab, 1∶25), β-actin (Sigma-Aldrich, 1∶10000) rabbit α-tubulin, detyrosinated (Millipore, 1∶500), rat α- tubulin YL1/2 (Abcam, 1∶5000) or mouse α-WT1 (DAKO, 1∶2000). For detection of KATNAL1 one western blot was probed with anti-KATNAL1 antibody following incubation with excess of the appropriate blocking peptides following manufacturer's instructions. Primary antibody was detected using goat anti-rabbit IRDye 800 (LI-COR, 1∶10,000), donkey anti-rat IRDye 800CW (Rockland Immunochemicals, Gilbertsville, PA, 1∶10,000) or goat anti-mouse IRDye 680 (LICOR, 10,000) respectively. Detection was carried out using the LI-COR Odyssey imaging system (LI-COR biosciences) according to manufacturer's instructions.

### Immunohistochemical analyses

Sections were deparaffinised, rehydrated and antigen retrieved before blocking of non-specific binding sites as detailed previously [47]. Sections were incubated overnight at 4°C with primary antibody diluted as described (Table S1). Diaminobenzidine (DAB) immunostained slides were counterstained with hematoxylin, dehydrated and mounted with Pertex (Histolab, Gothenberg, Sweden), and images captured using a Provis Microscope (Olympus) equipped with a Kodak DCS330 camera (Kodak, Rochester, USA). For Cleaved Caspase-3 detection, automated immunohistochemistry was performed on a Bond-max machine as previously described [48]. Fluorescent immunostained sections were detected using Tyramide 488, Tyramide 563 or direct alexa staining according to manufacturer's instructions, and counterstained with Propidium Iodide or Sytox Green. To ensure reproducibility of results representative testes from at least five animals at each age were used, and sections from fertile and infertile males were processed on the same slide on at least three occasions. For KATNAL1, control slides were generated following incubation of the KATNAL1 antibody with an excess of the appropriate blocking peptides according to manufacturer's instructions (Covalab, Cambridge, UK). For the other, well-characterised antibodies, incubation with normal serum alone in place of the primary antibody was used.

### Determination of testicular cell composition

Standard stereological techniques involving point counting of cell nuclei were used as previously described [46], [49] (n = 5 per genotype).

### Statistical analysis

Data were analysed using GraphPad Prism version 5 (Graph Pad Software Inc, San Diego, USA) using a 2-tailed Fisher's Exact Test; a 2-tailed unpaired *t* test; or a 1-way ANOVA, followed by Bonferroni post-hoc tests. Values are expressed as mean ± SEM.

## Supporting Information

Figure S1 Validation of a custom KATNAL1 antibody. The efficacy of the custom-designed KATNAL1 antibody was confirmed via dual-colour fluorescent Western blotting on whole-testis lysate. This identified a single, specific band corresponding to KATNAL1. An antibody against β-actin labelled with a different fluorophore was included as a loading-control antibody. (Note: KATNAL1 does not cross-react with β-actin, original dual-fluorescent image represented in grayscale to aid visualization).
(TIF)

Table S1 Antibodies and detection methodologies for immunohistochemical analysis. A summary of the suppliers, concentrations used and detection methodology for each primary antibody used for immunohistochemical analysis within the study.
(DOCX)
