# Supplementary figures and images for: KATNAL1 Regulation of Sertoli Cell Microtubule Dynamics Is Essential for Spermiogenesis and Male Fertility
Source: PLoS Genet. 2012 May 24;8(5):e1002697. doi: 10.1371/journal.pgen.1002697 (PMC3359976; doi:10.1371/journal.pgen.1002697)

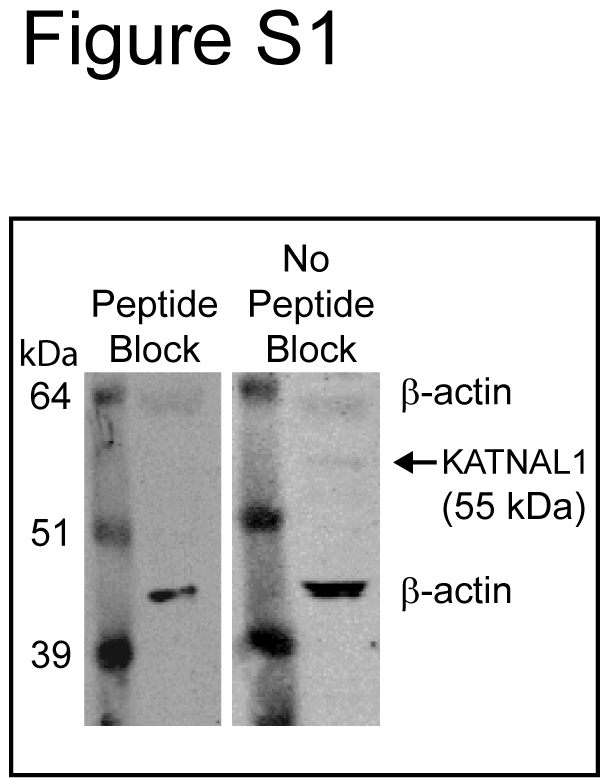

Supplement: Figure S1 — Validation of a custom KATNAL1 antibody. The efficacy of the custom-designed KATNAL1 antibody was confirmed via dual-colour fluorescent Western blotting on whole-testis lysate. This identified a single, specific band corresponding to KATNAL1. An antibody against β-actin labelled with a different fluorophore was included as a loading-control antibody. (Note: KATNAL1 does not cross-react with β-actin, original dual-fluorescent image represented in grayscale to aid visualization). (TIF) [file pgen.1002697.s001.tif]
