# Supplementary material for: KATNAL1 Regulation of Sertoli Cell Microtubule Dynamics Is Essential for Spermiogenesis and Male Fertility
Source: PLoS Genet. 2012 May 24;8(5):e1002697. doi: 10.1371/journal.pgen.1002697 (PMC3359976; doi:10.1371/journal.pgen.1002697)
Supplement: Table S1 — Antibodies and detection methodologies for immunohistochemical analysis. A summary of the suppliers, concentrations used and detection methodology for each primary antibody used for immunohistochemical analysis within the study. (DOCX) [file pgen.1002697.s002.docx]

**Table S1**

| Antibody | Source | Dilution | Detection system |
| --- | --- | --- | --- |
| TUBB3 | Sigma-Aldrich, Gillingham, UK | 1:2000 | Goat anti-mouse biotinylated + DAB |
| Cleaved-Caspase-3 | Cell Signalling Technology, Beverley, USA | 1:100 | described in [48] |
| KATNAL1  (Custom made) | Covalab, Cambridge,  UK | 1:300 | Tyramide |
| PGK1/2  Espin  α-tubulin, detyrosinated | Santa Cruz, Biotech, CA, USA  BD Transduction Laboratories  Millipore, Billerica, MA, USA | 1:800  1:100  1:3600 | Tyramide  Goat anti-mouse Alexa 406  Tyramide 563 |
|  |  |  |  |
